# The Appendix

**Published:** 1909-09

**Authors:** Theodore W. Nevin


### The Appendix.

Appendix, worm-like thing,
To thee I sadly sing;
My woe is great.
Thee I must bid good-bye
Or else I'll surely die,
So say grave surgeons *zwei,*
Quite up to date.

They tell me how 'tis done;
Gad! prove it's merely fun,
Yea, nothing more.
The skin is giv'n a slash,
Deft hands through muscles dash,
Thy neck's slit in a flash—
All's safely o'er.

Worm, true thou hast been,
Heldst fast through thick and thin,
In weal and woe;
"Blind process" though thou art,
Deemed worthless from the start,
I love thy every part—
Thou'rt not a foe.

Thou'st served me night and day,
In modest, humble way,
Through youth and age;

Hence I would ingrate be
To e'er go back on thee;
Not fit my God to see,
With such a page.

So then I'll here decide,
Thou shalt with me abide
For e'er and aye.
E'en tho they claim with heat
That thou'rt corruption's seat;
That's defeat's plaintive bleat,
Its dying cry.

—*Theodore W. Nevin, in Medical World.*

